# Molecular and epidemiological characterization of HIV-1 subtypes among Libyan patients

**DOI:** 10.1186/s13104-017-2491-2

**Published:** 2017-04-28

**Authors:** Mohamed A. Daw, Abdallah El-Bouzedi, Mohamed O. Ahmed, Aghnyia A. Dau, In association with the Libyan Study Group of Hepatitis & HIV

**Affiliations:** 1 Department of Medical Microbiology, Faculty of Medicine, University of Tripoli, CC 82668 Tripoli, Libya; 2 Department of Laboratory Medicine, Faculty of Biotechnology, University of Tripoli, CC 82668, Tripoli, Libya; 3 Department of Microbiology and Parasitology, Faculty of Veterinary, University of Tripoli, CC 82668 Tripoli, Libya; 4 Department of Surgery, Tripoli Medical Centre, Faculty of Medicine, University of Tripoli, CC 82668 Tripoli, Libya; 5 Tripoli, Libya; 6 Department of Medical Microbiology and Immunology, Faculty of Medicine, University of Tripoli, Tripoli, Libya

**Keywords:** HIV-1 subtypes, Libya, Genetic diversity, Molecular epidemiology, AIDS, BG-S-Benghazi–Bulgarian Strains

## Abstract

**Background:**

The epidemiological and clinical aspects of human immunodeficiency virus subtypes are of great interest worldwide. These subtypes are rarely studied in North African countries. Libya is a large country with the longest coast on the Mediterranean Sea, facing the Southern European countries. Studies on the characterization of HIV-1 subtypes are limited in Libya. This study aimed to determine the magnitude of the HIV problem among the Libyan population and to better understand the genetic diversity and the epidemiologic dynamics of HIV 1, as well as to correlate that with the risk factors involved.

**Methods:**

A total of 159 HIV-1 strains were collected from 814 HIV positive patients from the four Libyan regions during a 16-year period (1995–2010). To determine the HIV-1 subtypes, genetic analysis and molecular sequencing were carried out using provirus polygene. Epidemiologic and demographic information was obtained from each participant and correlated with HIV-1 subtypes using logistic regression.

**Results:**

The overall prevalence of HIV among Libyans ranged from 5 to 10 per 100,000 during the study period. It was higher among intravenous drug users (IVDUs) (53.9%), blood recipients (25.9%) and heterosexuals (17.6%) than by vertical transmission (2.6%). Prevalence was higher among males aged 20–40 years (M:F 1:6, *P* > 0.001). Among the 159 strains of HIV-1 available for typing, 117 strains (73.6%) were subtype B, 29 (18.2%) were CRF02_AG, and 13 (8.2%) were subtype A. HIV-1 subtype B was the most prevalent all over the country, and it was more prevalent in the Northern region, particularly among IVDUs (*P* < 0.001). GRF02_AG was common in the Eastern region, particularly among blood recipients while subtype A emerged in the Western region, particularly among IVDUs.

**Conclusions:**

HIV-1 infection is emerging in Libya with a shifting prevalence of subtypes associated with the changing epidemiology of HIV-1 among risk groups. A genetic analysis of HIV-1 strains demonstrated low subtype heterogeneity with the evolution of subtype B, and CRF_20 AG, as well as HIV-1 subtype A. Our study highlights the importance of expanded surveillance programs to control HIV infection and the necessity of introducing public health strategies to target the risk groups, particularly IVDUs.

## Background

The emergence of the acquired immunodeficiency syndrome (AIDS) among human populations has been traced back to between the 1900s and early 1920s, but it was not until 1983 that the human immunodeficiency virus (HIV) was identified as its cause. HIV belongs to the *Retroviridae* family, the Lentivirus genus. This genus comprises both types of HIV (HIV-1 and HIV-2), in addition to many simian immunodeficiency viruses (SIV) that naturally infect different primate species in Africa [1, 2]. HIV types have distinct patterns of spread and progression to AIDS. HIV-2 infection is mainly restricted to regions of Western and Central Africa and account for only 1.4% of HIV isolates [3]. HIV-1, which is responsible for the AIDS pandemic, has been divided into four groups (M, N, O and P), each of which is derived from a distinct introduction of simian immunodeficiency viruses that naturally infect chimpanzees (SIVcpz). HIV-1 group M (Major) alone is responsible for more than 95% of the AIDS pandemic, and virtually all studies on HIV have been conducted with representatives of this group [4, 5].

The epidemiological and clinical features of HIV infection have changed considerably in recent years. This is clearly mirrored by changing in the modes of transmission and evident drop in related morbidity and mortality [6, 7]. The trajectory of the AIDS epidemic has been broken in most regions, and sustainable solutions are implemented worldwide. However, despite all the progress, the AIDS epidemic is far from over, particularly in developing countries [8]. In those countries, which are rampant with inequalities, political instability, and discriminatory regulations, fragile communities affected by HIV and AIDS are found everywhere [9, 10]. Therefore, studies are needed to highlight the ever-changing epidemiology of HIV among the infected populations in such countries.

The global epidemiology of HIV-1 subtypes is very heterogeneous and varies greatly worldwide, even within regions of the same continent [11, 12]. During 2004–2007, subtype C accounted for nearly half (48%) of all global infections [4, 13]. It is considered endemic in sub-Saharan and Eastern Africa (where over two-thirds of infected individuals reside) followed by the Indian Pacific and southern region of Brazil. HIV-1 subtype A1 is more prevalent in Central Africa, Iran, Eastern Europe and Central Asia, as it accounts for 12% of the total subtypes. Subtypes A2 and A3 are found primarily in Africa. Subtype B is the most disseminated variant. It accounts for 11% and found mainly in the European Union, USA, Australia, North Africa and Japan [14, 15]. CRF02_AG accounts for 8% and CRF01_AE for 5%, and they occur mainly in Western Africa and Southeast Asia [2, 16]. Other CRFs and URFs are responsible for 4% of global infections, bringing the combined total of worldwide CRFs to 16% and all recombinants (CRFs plus URFs) to 20% [17]. Other subtypes, such as subtype G and D, account for 5 and 2%, respectively, while subtypes F, H, J and K together cause fewer than 1% of HIV infections worldwide [18, 19].

The HIV epidemic in North African countries is growing faster nowadays due to political instability, which resulted in massive population displacements and lack of migration control [20, 21]. Most transmission within these countries occurs among intravenous drug users (IVDUs) and in some health-care settings [22, 23]. Few studies have been reported on the genetic variability of HIV-1 in these countries. In Libya, epidemiological data on the HIV-1 epidemic is lacking [24, 25]. The geographic location of the country, with the longest coast in the Mediterranean basin facing the Southern European countries, and its oil producer status make it an important destination for Sub-Saharan immigrants [26, 27]. Hence, studying the HIV-1 subtypes in Libya should be a priority. The objectives of this study were to perform a comprehensive molecular epidemiological survey of HIV-1 diversity in Libya and to investigate the prevalence of viral variants circulating among different risk groups to better characterize the HIV-1 epidemic.

## Methods

### Study population

A total of 814 persons diagnosed with HIV-1 infection in Libya were studied over a 16-year period from 1995 to 2010 (Table 1). Convenience specimens were collected from different vulnerable populations, including blood and blood product recipients, IVDUs, offspring of infected mothers, and persons with promiscuous sexual contacts (Table 1). Venous blood samples were taken by qualified nurses and immediately tested for HIV by laboratory technicians who applied a three-serial rapid testing strategy using determine HIV-1/2 Kit (Inverness Medical Innovations) for initial testing, and subsequently Uni-Gold HIV-Kit (Trinity Biotech) and Bioline HIV-1/2-Kit (Standard Diagnostics) for confirmation of reactive results [29], The sera samples were collected from each of the four regions according to the Libyan Ministry of Housing & Planning, as recently described by Daw et al. [28]. Of the 814 infected samples, only 159 samples (19.5%) were found suitable for genotyping and subtype specification. Of these, 35 (22%) were from the Eastern Region, 42 (26.4%) from the Western Region, 59 (37.1%) from the Northern Region, and 23 (14.5%) from the Southern Region. Demographics and relevant clinical data were taken from the questionnaire database and cross-referenced with the patients’ medical files, as previously described [28].

**Table 1:** Distribution by age, gender, route of infection and year of diagnosis of HIV-1 seropositive patients in Libya

Demographic characteristics	Registered patients^a^		Ratio^b^
	Studied	Total	
Age (years)			
<20	49	119	1:2
20–40	87	623	1:7
>40	23	72	1:3
Gender			
Male	132	745	6:5
Female	27	69	1:3
Transmission route			
Blood or blood products	61	211	1:4
Sexual contact	17	143	1:9
IVDU	72	439	1:7
Vertical transmission	9	21	5:3
Screening period			
1995–2000	48	311	1:7
2001–2005	52	217	5:4
2006–2010	59	286	1:5
Total	159	814	1:5

^a^Officially registered and coded cases of HIV in Libya (1995–2010)

^b^Ratio (Studied: Registered)

### Genetic analysis

The HIV-1 genotyping and genetic analysis was carried out using partial PCR amplification was performed on a convenience sample of 159 collected from different patients all over Libyan regions. A multiple alignment of the newly derived protease/RT sequences and full-length genome sequences with selected reference sequences was constructed and the origin of the subtypes were traced and analysed as previously described [30]. Phylogenetic trees were generated, and the consistency of branching order was evaluated using Phylogeny Inference Package V3.5c. Recombinant analysis used Simplot, version 3.4,20 and alignment examination determined precise breakpoints. After breakpoint identification, each segment was extracted and analyzed phylogenetically to confirm the assignment of the subtype. Serologic test results were linked to the questionnaire only by a unique numeric code to preserve confidentiality and anonymity of the study participants [31].

### Statistical analysis

The data were coded and entered in an Excel sheet. The database was cleaned and verified. The data were analyzed by using Microsoft Excel 2007, and statistical tests were done using Minitab version 15 and SPSS version 16. A Chi square test was used to determine the difference in risk factors between seropositive and sero-negative viral status. Odds ratios (OR) and 95% confidence intervals (CI) in univariate analysis were calculated using seronegative persons as reference. Multiple logistic regression was used for multivariate analysis to determine the independent risk factors for exclusively all HIV-1 subtypes involved.

## Ethical considerations

The study was approved by the Libyan National Ethical Committee (Approval No. LY NS, AIDS-HIV-310153). It was conducted in accordance with the Helsinki Declaration [32] and under the supervision of the Faculty of Medicine, University of Tripoli, Tripoli, Libya. Clinical and epidemiological data were maintained only for therapeutic and research purposes. All participants signed an informed consent form witnessed by the local health officer before collection of data and blood samples. The questionnaire used to collect demographic and epidemiological data was anonymous and linked to the blood sample tube only by a code. This study was conducted in collaboration with both the Libyan Study Group of Hepatitis & HIV and the Libyan Society of Hospital Infection (LSHI). Both organizations have a strong human rights focus as well as extensive involvement in hepatitis and AIDS prevention activities in the region and consequently have managed to establish strong collaborative links with government, public institutions, and civil society.

## Results

A total of 814 HIV-seropositive cases from all the four regions of the country were followed. Of these, 745 (91.5%) were males (M:F ration 10.8:1). The largest number of infections were attributed to IVDUs (439, 53.9%) followed by those who had received blood or blood products (211, 25.9%), sexual contact (143, 17.6%) and mother-to-child transmission (21, 2.6%). The prevalence was significantly higher among males (M:F ratio 1:6, *P* < 0.001) and among those aged 20–40 years. It was lower among those <20 years old and those >40 years old (*P* > 0.01). The prevalence of HIV-1 among the studied population changed over the study period by a ratio (R) of 1:1.5 (1995‒2000 vs. 2005‒2010).

**Fig. 1:**
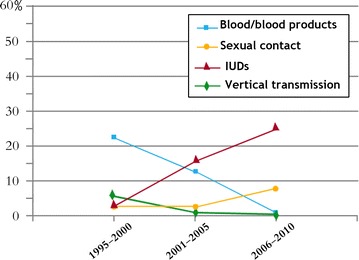
Prevalence of HIV-1 subtypes among risk groups in Libya 1995–2010

The trends in the prevalence of HIV infections among major risk groups during 1995‒2010 are shown in Fig. 1. During the early part of the study, HIV-1 prevalence was high among persons receiving blood or blood products and via vertical transmission (*P* < 0.001). These prevalence rates declined considerably by the end of the study period, possibly due to strict adherence to the universal obligatory blood screening program introduced in Libya in the early 1980s. On the other hand, the frequency of infected IVDUs increased significantly during the study period, from 2.5% during 1995‒2000 to 26.4% in 2005‒2010 (1:10.5). There was a slight increase in the frequency of those who contracted HIV via sexual behavior from 2.5 to 5.7% (R 1:2.3). The decline among those receiving blood products was considerable, from 21.4 to 0.5% (R: 4.3:1).

**Fig. 2:**
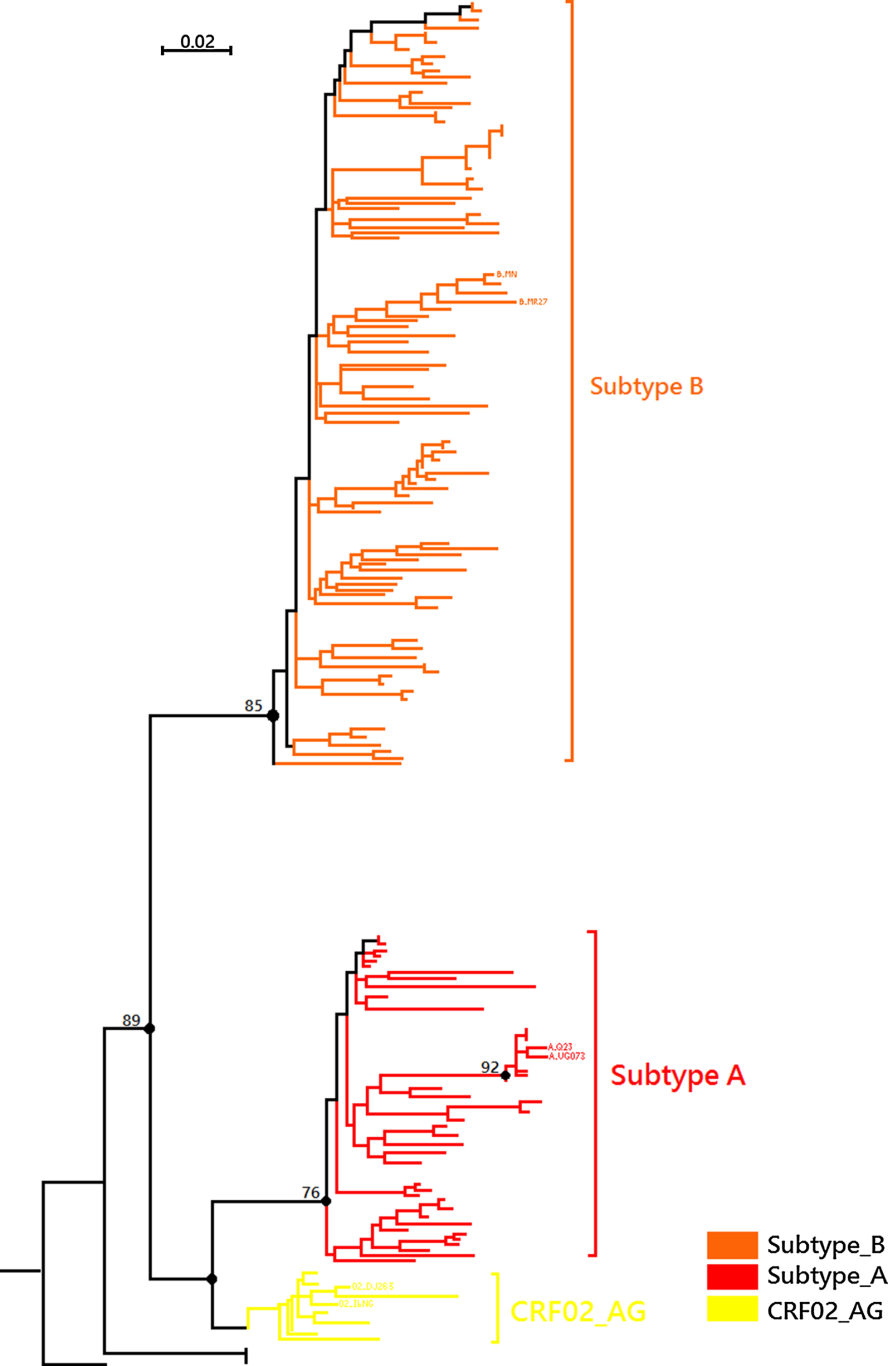
Phylogenetic tree built using the sequence analysis of 159 strains collected from all regions of Libya. Subtype B (*orange*), subtype A (*red*), and CRF02_AG (*yellow*). Reference samples are those named, preceded by the subtype

Neighbor-joining trees of the HIV sequences of the 159 available samples and reference sequences of HIV-1 group M (subtypes A, B, C, D, G, and CRF02_ A/G) revealed three distinct subtypes, including subtype A, subtype B and CRF02_AG. Figure 2 shows the phylogenetic tree built using the sequence analysis of 159 strains collected from all regions of Libya with reference samples are those named preceded by the HIV-1 subtype.

**Fig. 3:**
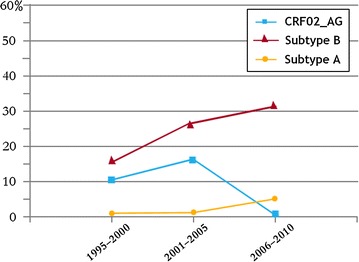
Distribution of HIV 1 subtypes among Libyan patients 1995–2010

Subtype B was the most frequent subtype, accounting for 73.5% of the samples, followed by CRF02_AG (18.3%). Subtype A accounted for 8.2%. According to patient retrieval data, all persons involved in this study were presumed to have been infected in Libya. The prevalence of the reported subtypes varied greatly over the study period as shown in Fig. 3. There were 16 CRF02_AG strains (10.1%) in 2000 but only five (3.1%) in 2010, whereas subtype B represented 16.4% in 2000 and reached 32.7% in 2010. However, the prevalence of a few strains of subtype A did not change significantly during the study period.

**Fig. 4:**
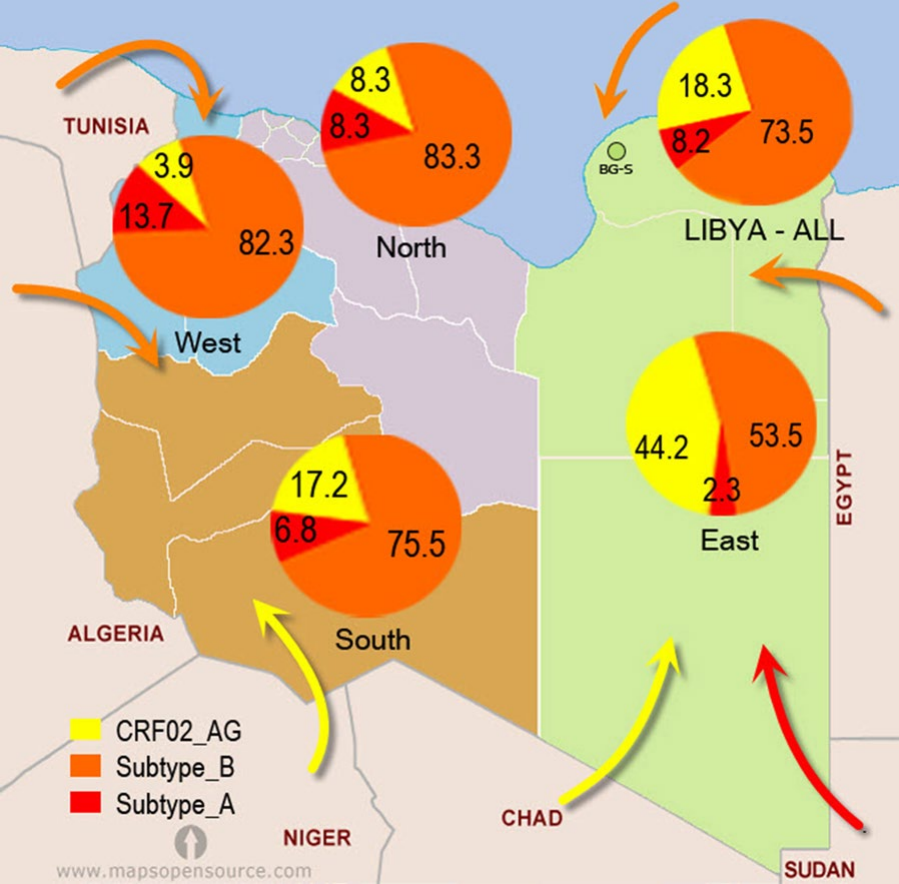
Map of Libya shows the distribution of HIV-1 subtypes among the Libyan regions. The arrows indicate the plausible route of HIV-1 subtypes entry into Libya, including BG-S (Benghazi‒Bulgarian Strain)

The association of HIV-1 subtypes with demographic and risk factors were analyzed. These included age, gender, mode of transmission, and time of diagnosis as shown in Table 2. HIV-1 CRF02_AG was most frequent among those who received blood or blood products, and among those <20 years old, followed by those who were infected by vertical transmission. Subtype B was more frequent among IVDUs, followed by those with promiscuous sexual contact predominantly among the sexually active group (20‒40 years). A few strains of subtype A were identified, mainly among IVDUs aged 20‒40 years. The proportions of younger persons infected with CRF02_AG declined significantly over the study period, whereas subtype B increased (*P* < 0.001), particularly among IVDUs and those practicing sexual behavior. There was a significant spike in the relative prevalence of subtype B during 2006–2010, while CRF02_AG declined (OR 4.1; *P* < 0.001). Table 3 shows the distribution of HIV-1 subtypes by region of residence. During 1995‒2000, 11 (57.9%) strains of CRF02_AG were isolated from the Eastern region, 5 (26.3%) strains from the South, 2 (10.5%) from the North, and only 1 (5.3%) from the West. Subtype B was predominant in the North (11; 47.8%) and West (7; 30.4%), and only a few strains were isolated from the East (3; 13%) and South (2; 8.7%). Only two strains of subtype A were emerging in the Western region. Between 2001 and 2010, the contribution of subtype B increased up to three folds in most of the regions. This was paralleled by a reduction of subtype CRF02_AG, particularly in the Eastern region, where it was the main subtype during 1995‒2000. However, a few strains of subtype A were emerging in the West and South. The location of the various genotypes was plotted on a map of Libya to examine the trends in their geographic distribution as illustrated in Fig. 4. The arrows on the map indicate the plausible route through which these strains may have entered Libya, including the Benghazi‒Bulgarian strains (BG-S). Most infections and the greatest heterogeneity was observed all over the country, but they particularly more heterogeneous in the Northern region, where the capital Tripoli is located, followed by the three other regions. The most common B subtypes showed the widest distribution across the country. Most CRF02_AG cases were located in and around Benghazi in the Eastern region of Libya.

**Table 2:** Association between infection with different HIV-1 subtypes and certain influencing variables among the Libyan population

	CRF02_AG			Subtype B			Subtype A		
	OR	95% CI		OR	95% CI		OR	95% CI	
		Lower	Upper		Lower	Upper		Lower	Upper
Age group (years)									
<20	2.3	1.34	2.62	0.60	0.35	0.95	0.0	0.0	0.0
21–40	0.20	0.09	0.24	2.89	1.95	3.95	0.70	0.43	1.02
>40	0.0	0.0	0.0	2.91	2.21	3.9	0.59	0.39	0.91
Sex									
M vs. F	0.75	0.63	0.89	1.71	1.68	1.98	1.62	1.46	1.79
Risk factors									
Blood transfusion	1.22	1.12	1.87	0.3	0.12	0.34	0	0	0
IVDU	0.0	0.0	0.0	6.31	2.15	17.15	5.65	1.75	17.06
Sexual contact	0.0	0.0	0.0	1.21	1.05	1.43	0	0	0
Vertical transmission	0.9	0.8	1.90	0	0	0	0	0	0
Screening period									
1995–2000	1.06	0.8	2.4	1.9	0.6	3.4	0.0	0.0	0.0
2001–2005	0.02	0.01	1.02	2.7	1.2	5.6	0.01	0.01	0.01
2006–2010	0.01	0.0	0.42	4.1	2.2	6.8	0.01	0.01	0.04

*OR* odds ratio, *CI* confidence interval, *M* males, *F* females, *IVDU* intravenous drug use

**Table 3:** Distribution of HIV-1 subtypes by regions of residence among Libyans infected with HIV 1995‒2010

Region	1995–2000			2001–2005			2006–2010		
	HIV 1 subtypes								
	CRF02_AG	A	B	CRF02_AG	A	B	CRF02_AG	A	B
East	11	0	3	3	0	5	2	0	11
West	1	2	7	1	2	10	0	3	16
North	2	0	11	1	2	17	0	1	25
South	5	0	2	2	0	3	1	3	7
Total	19	2	23	7	4	35	3	7	59

## Discussion

Despite the overwhelming progress in understanding the global epidemiology of HIV, few accurate data are available on the prevalence of HIV-1 infection in North African countries. Studies in this region have been hampered by fears of stigma and discrimination against people living with HIV/AIDS [33]. In Libya, a few studies were carried out on the prevalence of co-infection with HIV and hepatitis B or hepatitis C virus, but no study has focused on the magnitude of the HIV problem in the Libyan population [29]. Consequently, we performed a comprehensive cohort study over a 16-year period of individuals newly diagnosed with HIV-1 from all geographical regions of Libya. The overall prevalence of HIV-1 in our study ranged from 5 to 10 per 100,000 according to the cases registered in Libya. Furthermore, the prevalence varied from one risk group to another. It was highest among IVDUs, followed by blood recipients, and to a lower extent people with promiscuous sexual behavior. In our study, the number of people infected via IVDU increased steadily from the late 2000 and doubled by the end of 2010. Most cases newly infected with HIV were among IVDUs, only 10% were attributable to heterosexual exposure, and rarely to other risk factors. This is in agreement with other studies conducted in the Middle East and North Africa (MENA) region. There is documented evidence of concentrated epidemics of HIV among IVDUs in Egypt, Morocco and Algeria, and suggestive evidence of epidemics among heterosexuals in Sudan and Egypt [34, 35]. However, most infections in these countries seem to be concentrated in high risk groups and there is no evidence of a sustainable general population epidemic. Therefore, large-scale cross-sectional studies are needed to verify that assumption, with further validity assessments for extrapolations in these countries [36, 37].

Molecular analysis of HIV-1 subtypes among Libyan patients showed three distinct subtypes. Subtype B was the most prevalent genotype, representing over 70% of HIV-1 infections in Libya, followed by CRF02_AG (18.3%) and subtype A (8.2%). This is in an agreement with other studies from neighboring Northern African countries. In Egypt, subtype B was the commonest strain, accounting for 91.7% of cases, followed by O (4.2%) and CRF01_AE (4.2%) [38]. In Tunisia, subtype B accounted for 95.8% and CRF02_AG for 4.2% [39]. Similarly, in Morocco HIV 1-subtype B accounted 93.5%, subtype A for 1%, and subtype F for 0.5% [40]. In Algeria, subtype B accounted for 56.3%, followed by CRF02_AG (18.0%), along with other contributing strains (CRF06_cpx 17.2, G 2.7, 206 2.7, D 1.3, and others 1.9% [41]. CRF02_AG and subtype A strains were also common in Sub-Saharan and West and Central Africa. However, the CRF02_AG strains found in our study were monophyletic, with low genetic diversity, and so are likely descended from a common ancestor [42, 43]. Similar results were reported from European countries in the Mediterranean basin, particularly Italy, Spain and Greece, with local expansion in certain populations, particularly IVDUs and heterosexuals [44, 45].

The reasons for the expansion and spread of HIV-1 subtypes in Libyan regions are not clear, and it is difficult to trace the possible evolutionary history of the Libyan subtypes. Among the speculative factors are immigration, scandals and population integration within sub-Saharan and North African countries [36, 46]. The substantial and continuous increase in subtype B strain among Libyans observed in this study could be attributed to the large number of legal and illegal workers who come to Libya from Egypt and neighboring Maghreb countries, where this strain is dominant [28, 29]. In the Eastern region of Libya in 1998, a unique circulating form was suddenly identified among 400 children with no known previous exposure or risk factors who had been treated at Benghazi Pediatric Hospital in Benghazi [25]. There is an evident speculation that this was caused by an unannounced testing of an attenuated vaccine strain by foreign medical staff, similar to vaccine trials early last century, in which children from developing countries were used [47–49]. In any case, HIV 1 subtypes distribution in Libya shows limited diversity, and further epidemiological studies are needed to clarify the overlapping chains of transmission across North African countries.

The prevalence of HIV 1 subtypes among risk groups varies greatly from one part of the world to another. In Europe and North America, subtype B was found to be endemic among IVDUs, but in Cambodia and Vietnam subtype E was the most common [50, 51]. In our study, the initial epidemic among IVDUs was largely due to subtype B, whereas subtype CRF_02 AG was more prevalent among blood product recipients and a those infected by vertical transmission. Subtype A emerged among all risk groups, particularly those prone to promiscuous sexual contacts. This is in agreement with other studies from Africa, USA and EU on subtype B, though CRF_20AG and subtype A were common among Sub Saharan African countries [49, 52].

On the population level, none of our patients had multiple infections with HIV-1 subtypes. This observation of low inter-patient diversity has also been reported in epidemics of HIV-1 subtypes in the former Soviet Union and Ukraine [53]. Furthermore, differences in pathogenicity among HIV-1 genetic subtypes were not noted in the current study. However, Zhang et al. [54] reported higher initial viral loads, with similar CD4 cell counts in patients with subtype CRF_01AE and patients with subtype B. Thus, further studies are needed to evaluate antiretroviral therapy of HIV-infected patients in Libya.

Despite the dominance of subtype B in the Libyan population, the prevalence of subtypes is both periodically and geographically variable. CRF_02 AG, which was associated mainly with blood products, was more prevalent at the start of the study (1995) but was rarely reported by the end of the study (2010). This subtype was dominant mainly in the Eastern region of the country, where it accounted for over 45% of HIV-1 infection followed by the Southern region (17%), but it was rarely reported in the other regions (<8%). This was not the case for subtype B, the prevalence of which rose significantly throughout the study period, particularly among IVDUs and all over the Libyan regions, but particularly in West and South regions. Subtype A first appeared in 2005 and its prevalence increased, particularly in the Western and Northern regions.

It has been suggested that mobile populations play a special role in HIV-1 epidemics, particularly in developing countries. HIV-1 prevalence in communities located on commercial transport routes in Africa was found to be higher than that in communities elsewhere in the same region [55]. Libya is considered a major transit area of migration from Sub-Saharan Africa towards Western Europe for people seeking better living conditions. Subtype CRF02_AG and subtype A has been found to be endemic in Sub-Saharan Africa. Epidemiological studies have shown that HIV-1 Subtype A and CRF02_AG represent approximately 27% of HIV-1 infections worldwide, most of which are in Sub-Saharan Africa; CRF02_AG alone represents 50–70% of HIV-1 infections in this region. Thus, it could be easily introduced into Libya by immigrants. Although these indicators cause concern, no systematic information is available on the extent of the HIV-1 epidemic among African immigrants and on its behavioral determinants [56]. Such information is necessary to develop data-driven prevention programs.

The presented data give clear insights into the epidemiology HIV-1 among Libyans, which has hardly been explored in previous studies [29, 47]. However, the main limitation in our study is that it was carried out on registered HIV patients and so it may not mirror the exact picture of HIV in the country, as many HIV infected individuals may not get registered in the HIV national registry. Thus, speculation could be raised about the possibility that the overall prevalence masks an ongoing dynamic of HIV-1 among Libyan populations. Therefore, the trends need to be verified through a national surveillance program for a longer time, and further studies are needed to highlight the challenges that have to be met in order to reduce the disease burden in Libya [28, 57].
